# Identification and Characterization of GPCRs for Pyrokinin and CAPA Peptides in the Brown Marmorated Stink Bug, *Halyomorpha halys* (Hemiptera: Pentatomidae)

**DOI:** 10.3389/fphys.2020.00559

**Published:** 2020-05-29

**Authors:** Seung-Joon Ahn, Jacob A. Corcoran, Robert K. Vander Meer, Man-yeon Choi

**Affiliations:** ^1^USDA Agricultural Research Service, Horticultural Crops Research Laboratory, Corvallis, OR, United States; ^2^Department of Biochemistry, Molecular Biology, Entomology and Plant Pathology, Mississippi State University, Starkville, MS, United States; ^3^USDA Agricultural Research Service, Biological Control of Insects Research Laboratory, Columbia, MO, United States; ^4^USDA Agricultural Research Service, Center for Medical, Agricultural, and Veterinary Entomology, Gainesville, FL, United States

**Keywords:** neuropeptide, GPCR, *pyrokinin*, CAPA, *Halyomorpha halys*

## Abstract

The brown marmorated stink bug, *Halyomorpha halys*, is an invasive hemipteran that causes significant economic losses to various agricultural products around the world. Recently, the *pyrokinin* and *capa* genes that express multiple neuropeptides were described in this species. Here we report six *pyrokinin* and *capa* GPCRs including two splice variants, and evaluate their (a) ability to respond to neuropeptides in cell-based assays, and (b) expression levels by RT-PCR. Functional studies revealed that the *H. halys* pyrokinin receptor-1 (HalhaPK-R1a & b) responded to the pyrokinin 2 (PK2) type peptide. RT-PCR results revealed that these receptors had little or no expression in the tissues tested, including the whole body, central nervous system, midgut, Malpighian tubules, and reproductive organs of males and females. HalhaPK-R2 showed the strongest response to PK2 peptides and a moderate response to pyrokinin 1 (PK1) type peptides (= DH, diapause hormone), and was expressed in all tissues tested. HalhaPK-R3a & b responded to both PK1 and PK2 peptides. Their gene expression was restricted mostly to the central nervous system and Malpighian tubules. All PK receptors were dominantly expressed in the fifth nymph. HalhaCAPA-R responded specifically to CAPA-PVK peptides (PVK1 and PVK2), and was highly expressed in the Malpighian tubules with low to moderate expression in other tissues, and life stages. Of the six GPCRs, HalhaPK-R3b showed the strongest response to PK1. Our experiments associated the following peptide ligands to the six GPCRs: HalhaPK-R1a & b and HalhaPK-R2 are activated by PK2 peptides, HalhaPK-R3a & b are activated by PK1 (= DH) peptides, and HalhaCAPA-R is activated by PVK peptides. These results pave the way for investigations into the biological functions of *H. halys* PK and CAPA peptides, and possible species-specific management of *H. halys*.

## Introduction

Neuropeptides are the largest group of insect hormones. They regulate or modulate a variety of physiological actions, such as fat body homeostasis, feeding, digestion, excretion, circulation, reproduction, metamorphosis, and behavior throughout all life stages (Nässel and Winther, 2010; Nässel and Zandawala, 2019). The PRXamide peptide family (X, a variable amino acid) is a well-characterized neuropeptide component having a common amino acid sequence, PRX-NH_2_, at the C−terminal end, which is conserved across Insecta (Jurenka, 2015). The peptides are classified into three subfamilies, each having diverse biological roles in insects: (1) pyrokinin (PK) includes the pheromone biosynthesis activating neuropeptide (PBAN) and the diapause hormone (DH), (2) the capability (CAPA) peptides (peptides produced from the *capa* gene), and (3) the ecdysis−triggering hormone (ETH).

The neuropeptides activate specific G protein-coupled receptors (GPCRs) that are involved in essential biological processes. Data mining and computational analyses of the *Drosophila* genome identified a variety of GPCRs for PRXamide peptides that are orthologous to the vertebrate neuromedin U receptor (NmU-R) (Hewes and Taghert, 2001; Vanden Broeck, 2001). The hypothesis of ligand-receptor coevolution was supported when GPCRs of *Drosophila* PRXamide peptides were first identified and found to be related to the vertebrate peptide neuromedin U (NmU) (Howard et al., 2000; Park et al., 2002). Since then, GPCRs for PRXamide peptides have been studied in other insect groups, and the first PBAN receptor was identified from *Helicoverpa zea* (Choi et al., 2003), followed by *Bombyx mori* (Hull et al., 2004). To date, many GPCRs for PBAN/PK2, DH/PK1, CAPA, and ETH peptides have been identified, characterized, and/or functionally expressed from various animal groups (reviewed by Jurenka, 2015).

The first CAPA peptides in hemipteran species were reported from the southern green stink bug, *Nezara viridula* (Predel et al., 2006). Later, various CAPA and PK peptides were identified from *Rhodnius prolixus*, a blood−feeding disease vector (Paluzzi et al., 2008; Predel et al., 2008; Paluzzi and Orchard, 2010; Ons, 2017), and the bed bug, *Cimex lectularius*, a global human ectoparasite (Predel et al., 2018). The GPCRs for CAPA and PK1 peptide variants were found to regulate the antidiuretic process in *R. prolixus* (Paluzzi et al., 2010; Paluzzi and O’donnell, 2012).

*Halyomorpha halys* is an invasive insect native to East Asia which has subsequently spread to Europe, North America and South America over the last couple of decades. This insect has become a serious polyphagous agricultural pest, using its proboscis to pierce and suck fluids from the stems of various crops, which in turn affects the plants’ growth rates, fruit production and esthetics (Leskey and Nielsen, 2018). Current control measures are based on integrated pest management tactics, using the recently identified aggregation pheromone (Khrimian et al., 2014) for mass trapping, and native and exotic parasitoids for biological control (Herlihy et al., 2016). More recent studies have taken molecular approaches, using RNA interference technologies to control these sap sucking insects (Ghosh et al., 2018).

We recently identified two *H. halys* PRXamide genes: a *pyrokinin* gene encoding three PK2 peptides (PK2-1, PK2-2, and PK2-3), but no PK1 (= DH) peptide; and the *capa* gene encoding two periviscerokinin (PVK) peptides (CAPA-PVK1 and CAPA-PVK2) and a CAPA-DH (= PK1-like) peptide (Ahn and Choi, 2018). In the present study, we report the identification of six GPCRs including two variant receptors associated with these peptides. Receptor gene structures determined, their expressions in different tissues and developmental stages were evaluated, and their responses to *H. halys* PK and CAPA peptides were characterized *in vitro*.

## Materials and Methods

### BLAST Search for PK and CAPA Receptor Sequences

Putative *H. halys* PK and CAPA receptors were identified via BLAST analysis of *H. halys* genomic and transcriptomic databases available through the i5k Workspace Project of the United States Department of Agriculture National Agriculture Library^1^ using the sequences of PRXamide receptors from other insects as queries.

### Cloning of PK and CAPA Receptor Genes

Total RNA was isolated from various tissues of male and female *H. halys*, and cDNA was synthesized using the Superscript IV First-Strand Synthesis Kit (Thermo Fisher Scientific, Waltham, MA, United States) using random hexamers and oligo-dT primers as described previously (Ahn and Choi, 2018). Primers (Supplementary Table S1) were designed using the Primer 3 application within Geneious v8.1.5 software (Biomatters, Ltd., Auckland, New Zealand). The primers were used to amplify full-length HalhaPK/CAPA-Rs from cDNA using Phusion High-Fidelity DNA polymerase (Thermo Fisher Scientific). PCR conditions were as follows: an initial incubation at 98°C for 2 m, followed by 35 cycles of 98°C for 10 s, 60°C for 15 s, and 72°C for 90 s, followed by a final 10 m extension at 72°C. PCR products were electrophoresed on a 0.7% agarose gel, and fragments of the expected size for each target were excised from the gel and purified using the GeneJet Gel Extraction Kit (Thermo Fisher Scientific). Following the manufacturer’s instructions, purified PCR products were inserted into the pIB/V5-His TOPO expression vector (Thermo Fisher Scientific) which allows for *OpIE2*-driven constitutive expression of the gene of interest in various insect cell lines. Bacterial colonies were tested for the presence of modified pIB plasmids by PCR using DreamTaq polymerase (Thermo Fisher Scientific) and gene-specific primers to amplify full-length sequences using the following PCR conditions: an initial 8 min incubation at 94°C followed by 30 cycles of 94°C for 30 s, 60°C for 30 s, and 72°C for 90 s. Three positive colonies were each inoculated into LB media and grown overnight. Plasmids were purified from bacterial cultures using the GeneJet Plasmid Miniprep Kit (Thermo Fisher Scientific) and Sanger sequenced to confirm the correct sequence and orientation of each gene within the pIB vectors. One plasmid containing each HalhaPK/CAPA-R gene with the correct sequence and orientation was chosen for transfection of insect cell lines.

### Expressing Receptors in Sf9 Cells

Five micrograms of each purified pIB/HalhaPK-R (or CAPA-R) plasmid and 15 μL of Cellfectin II reagent (Thermo Fisher Scientific) were diluted into 500 μL of serum free Insectagro Sf9 medium (Corning, Corning, NY, United States) and allowed to incubate for 10 min at room temperature, after which they were mixed together and allowed to incubate for an additional 15–20 min. Each pIB/HalhaPK-R (or CAPA-R) Cellfectin mixture was then added to T-25 cell culture flasks containing Sf9 cells at 50% confluency and incubated overnight at 28°C. The following morning the media was removed from each flask and replaced with media containing 20 μg/mL blasticidin (Corning). Each transfected cell line was cultured in the presence of 20 μg/mL blasticidin for ∼3 weeks until a blasticidin-resistant cell line was established, after which the blasticidin concentration was reduced to 10 μg/mL and the cell lines were frozen prior to testing in functional assays.

### Preparation of PK and CAPA Peptides

Peptides encoded by *H. halys pyrokinin* and *capa* genes used in this study have been recently identified (Ahn and Choi, 2018). Four peptides (>95% purity): PK2 (FYAPFSPRLa), CAPA-PVK1 (DAGLFPFPRVa), CAPA-PVK2 (EQLIPFPRVa), and CAPA-DH (= PK1-like, NGASGNGGLWFGPRLa) were synthesized to contain an amide group on their C-termini (Peptide 2.0 Inc., Chantilly, VA, United States). Peptides were solubilized in water, aliquoted into 2–20 nM working stocks, dried using a DNA120 SpeedVac Concentrator (Thermo Scientific), and frozen at –20°C until use in binding assays.

### Functional Assays of *H. halys* GPCRs

For each cell line expressing a specific *H. halys* PRXamide receptor, cells were thawed and cultured for at least three passages in a suspension culture containing blasticidin prior to functional assays. The transfected cell lines were grown as 20 mL suspension cultures in 50 mL-Erlenmeyer flasks with a screw cap, and shaken at 145 rpm at 28°C. For functional assays, cells were taken from a suspension culture in the exponential growth phase, and ∼50,000 cells were plated into each well of a tissue culture-treated, black-walled, 96-well plate (Corning). The plated cells were then incubated at 28°C for 48 h, after which the media was removed from the plates and the wells were rinsed with serum-free media, then loaded with 95 μL of 1X FLIPR Calcium 6 assay reagent (Molecular Devices, Sunnyvale, CA, United States). Plates were incubated at room temperature for 60 min then transferred to a FlexStation 3 Multi-Mode Microplate Reader (Molecular Devices) to measure cell fluorescence intensity from intracellular calcium influx after peptide-induced receptor activation. Wells of cells were then treated with 5 μL of water or 20X concentrations of each peptide using the automated injection system of the FlexStation plate reader. In all experiments, fluorescence was measured every 5 s for 30 s prior to treatment and for 2 min after exposure. In all cases, the maximum response obtained occurred ∼20 s after exposure to ligand. The mean response of four wells receiving the same treatment was calculated and expressed as a change in fluorescence from baseline (prior to treatment) to maximum response. For initial screening, each cell line was exposed to a single concentration of 500 nM of each peptide or control. Compounds that elicited responses were tested further in concentration-response experiments. Concentration-response curves and EC_50_ values were generated using the variable slope, four parameter non-linear regression function of GraphPad Prism data analysis (GraphPad Software, Inc., San Diego, CA, United States). Each cell line was tested for its response to peptides in at least three independent experiments.

### Reverse Transcriptase (RT) and Quantitative (q) PCRs

The following tissues: central nervous system, midgut, Malpighian tubules, testis and ovary, were dissected from 5 to 10 days-old adults and the total RNA was extracted as described previously (Ahn and Choi, 2018). cDNA was synthesized using the Superscript IV First-Strand Synthesis Kit (Thermo Fisher Scientific) using random hexamers and oligo-dT primers from 1,000 ng of the previously isolated RNA. Primers (Supplementary Table S1) were designed with the Primer 3 application within Geneious v8.1.5 software (Biomatters) and were used to amplify (a) full-length glyceraldehyde 3-phosphate dehydrogenase (GAPDH) as a control, and (b) HalhaPK/CAPA-Rs from cDNA using Phusion High-Fidelity DNA polymerase (Thermo Fisher Scientific). PCR conditions were as follows: initial incubation at 98°C for 2 m, followed by 35 cycles at 98°C for 10 s, 60°C for 15 s and 72°C for 90 s, then a final 10 m extension at 72°C.

Samples were collected from the following developmental stages: eggs, 1st to 5th nymphs, adult males and adult females (three biological replicates, each). Total RNA isolation, cDNA synthesis, and quantitative real-time PCR (qRT-PCR) were performed as described previously (Ahn and Choi, 2018). Briefly, cDNA was synthesized from 1,000 ng of total RNA using the Verso cDNA Synthesis Kit (Thermo Fisher Scientific) according to the manufacturer’s instructions. A qRT-PCR reaction mixture was prepared in 20 μL with 1,000 ng of cDNA, PowerUp SYBR Green Master Mix (Thermo Fisher Scientific), 0.25 μM of each primer, and RNase-free water. The reaction was run at 95°C for 15 min, then 40 cycles at 95°C for 15 s, and 60°C for 1 min using StepOnePlus^TM^ Real-Time PCR System (Applied Biosystems). Amplification specificity was followed by melting curve analysis over a 60–95°C range. Gene expression levels for the developmental stages were compared using *rpn2* as a reference gene.

## Results

### *H. halys* Receptor Identification, Cloning, and Sequence Comparison

**FIGURE 1:**
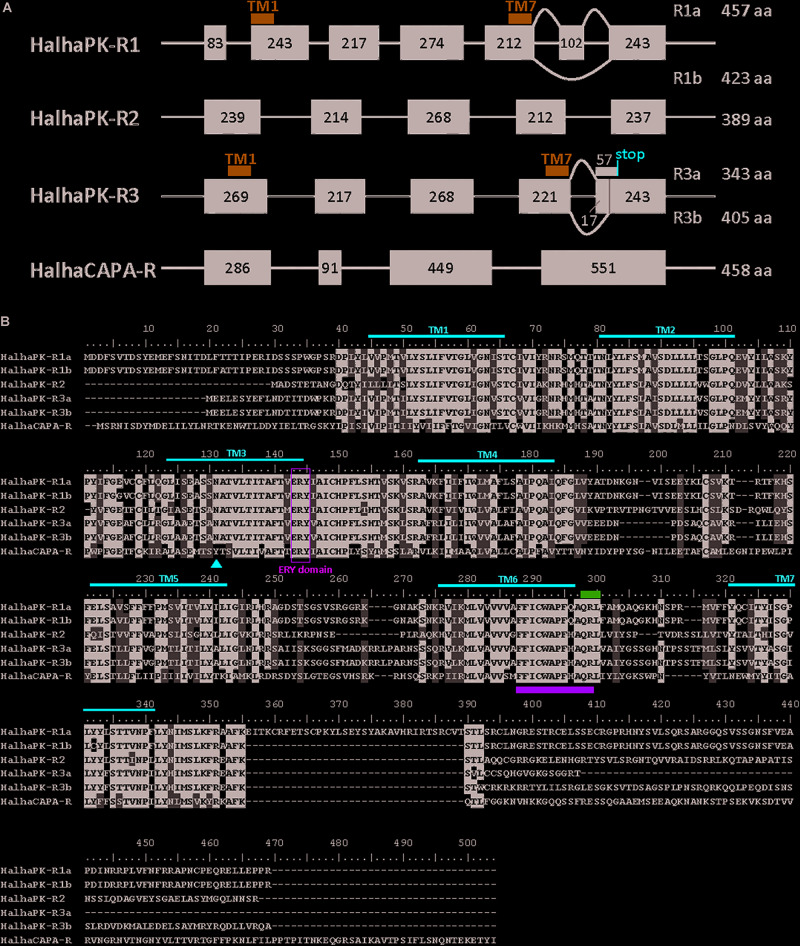
PRXamide receptor sequences from *Halyomorpha halys*. **(A)** Schematic genome structure of the four PRXamide receptors. Black boxes represent exons in coding sequences with the length of nucleotides inside the box. HalhaPK-R1 and HalhaPK-R3 produce two transcript variants (a,b), presumably by alternative splicing. Positions of the first and last transmembrane (TM) domains (TM1 and TM7) are indicated by blue bars only in HalhaPK-R1 and HalhaPK-R3, showing that the splicing occurs after TM7. **(B)** Alignment of the deduced amino acid sequences from the six receptors. Sequences were aligned using MUSCLE algorithm in MEGA 7.0. Consensus sequences were shaded with 70% cut-off using BioEdit. Seven transmembrane (TM) domains (red lines) were predicted by TOPCONS (Tsirigos et al., 2015). ERY domain (green box) and QRL motif (pink bar) are conserved in PRXamide receptors, and the rotamer toggle switch (green bar) is conserved in GPCRs in general. The red triangle in TM3 indicates a receptor-specific amino acid position in the PK receptors with N, and CAPA receptor with Y.

Six GPCRs for PRX family peptides were identified in *H. halys* (Figure 1A). Five of these were classified as *Halyomorpha halys* PK receptors (HalhaPK-Rs). These were designated as HalhaPK-R1a, HalhaPK-R1b, HalhaPK-R2, HalhaPK-R3a, HalhaPK-R3b, and one, HalhaCAPA-R, as a CAPA receptor. Sequences of the six receptors were aligned and their transmembrane domains determined (Figure 1B).

HalhaPK-R1 was present in the i5k genomic database on contigs XM_014437522.1 and HHAL010714-RA, and was comprised of 7 exons spread across a 56,938 bp region of scaffold2666. HalhaPK-R1 had two transcript variants with one comprised of all 7 exons (HalhaPK-R1a) and one missing exon 6 (HalhaPK-R1b). In addition, there were 6 non-synonymous mutations between the two versions of the proteins, which were validated by independent PCR methods and their presence in the genome. In the genome, some of the exons code for the HalhaPK-R1a transcripts and some code for HalhaPK-R1b transcripts, indicating that the genome sequence is an amalgamation of different HalhaPK-R1 alleles that were present in the pooled DNA used to generate the genome. HalhaPK-R2 was comprised of 5 exons spread across a 99,734 bp region of scaffold629, and was found as a complete transcript in contigs XM_014427306.1 and XM_014427307.1 in the i5k database. HalhaPK-R2 was amplified via PCR and the sequence of the resulting product exactly matched that of the transcriptome and genome. HalhaPK-R3a and HalhaPK-R3b were both present in the i5k genome and were comprised of 5 exons spread across a 56,025 bp region of scaffold1013, with two alternatives of exon 5. Both HalhaPK-R3a and HalhaPK-R3b were present in the i5k transcriptome on contigs XM_014431884.1 and XM_014431883.1, respectively. HalhaCAPA-R was present in the i5k genomic database, comprised of 4 exons spread across scaffold1099 (exons 1, 2 and 4) and scaffold3190 (exon 3), and in the transcriptome spread across two un-assembled contigs (XM_014432674.1 and XM_014437682.1). The HalhaCAPA-R transcriptomic and genomic sequences were identical.

### Functional Testing of GPCRs

**FIGURE 2:**
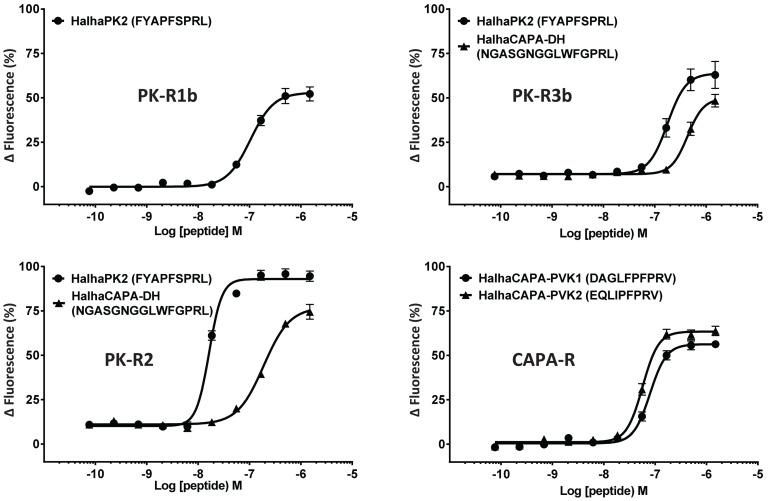
Concentration-response profiles of *Halyomorpha halys* receptors to pyrokinin and CAPA peptides that elicited responses in screening experiments. Data represent the mean ± SEM response of cells from three independent experiments. EC_50_ values for all six GPCRs are in Table 1.

**FIGURE 3:**
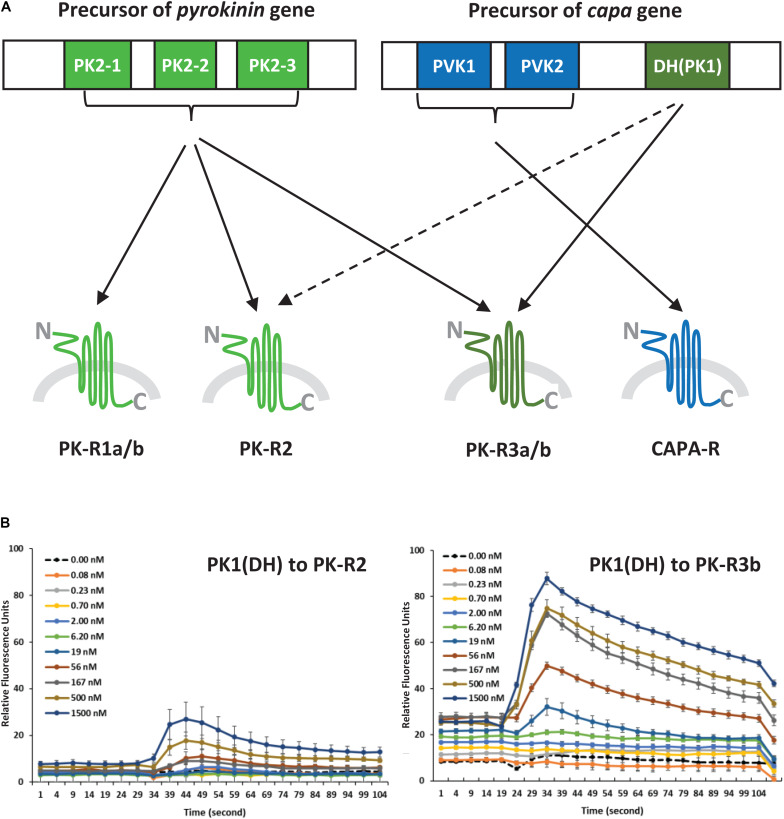
**(A)** Schematic diagram of ligand-receptor pairings determined by the concentration-response profiles of pyrokinin and CAPA peptides on *Halyomorpha halys* receptors. The colors indicate three groups of the ligand and receptor types, and the broken line indicates a weak response. **(B)** Responses of *H. halys* receptor 2 (PK-R2) and receptor 3b (PK-R3b) to various doses of CAPA-PK1(DH) over time. Data are representative, showing the mean ± SEM response of four wells of cells in a single experiment. Experiments were repeated on three occasions using naïve cells and fresh reagents.

In initial screening experiments, empty Sf9 cells and Sf9 cells expressing each of the six GPCRs were tested for ligand-induced receptor activation using each of the *H. halys* peptides or a negative vehicle control (Supplementary Figure S1). Empty Sf9 cells did not respond to any of the compounds tested. HalhaPK-R1a, -R1b, -R2, -R3a, and -R3b receptors all responded to PK2 and CAPA-DH peptides to varying degrees. HalhaPK-R2 responded strongly to PK2 peptides. HalhaPK-R3a and -R3b responded to PK1 (= CAPA-DH) and PK2 peptides, with the highest sensitivity to PK1 (= CAPA-DH), especially for HalhaPK-R3b (Table 1 and Figures 2, 3). HalhaCAPA-R responded specifically to PVK1 and PVK2 peptides (Table 1 and Figures 2, 3).

**TABLE 1:** Half-maximum effective concentrations (EC_50_) of the peptides tested on heterologously-expressed PK/CAPA receptors of *Halyomorpha halys*.

Peptide	Sequence	HhalPK-R1a	HalhaPK-R1b	HalhaPK-R2	HalhaPK-R3a	HalhaPK-R3b	HalhaCAPA-R
PBAN/PK2	FYAPFSPRLa	>1 μM	420 nM	35 nM	199 nM	142 nM	>1 μM
CAPA-DH/PK1	NGASGNGGLWFGPRLa	>1 μM	>1 μM	800 nM	660 nM	91 nM	>1 μM
CAPA-PVK1	DAGLFPFPRVa	>1 μM	>1 μM	>1 μM	>1 μM	>1 μM	80 nM
CAPA-PVK2	EQLIPFPRVa	>1 μM	>1 μM	>1 μM	>1 μM	>1 μM	57 nM

**“a” to indicate amide.**

Peptides that activated HalhaPK-Rs in initial screening experiments were subsequently tested in concentration-response experiments. GPCRs that responded to PK2 peptides in screening experiments showed positive concentration-dependent activity, with varying sensitivities and degrees of absolute magnitude. Similarly, receptors HalhaPK-R2, -R3a, and -R3b showed concentration-dependent responses to PK1 (= CAPA-DH), with varying sensitivities and degrees of absolute magnitude (Table 1 and Figure 2). HalhaCAPA-R had a concentration-dependent response to PVK1 and PVK2 (Figure 2), with similar sensitivity and degree of absolute magnitude.

### Tissue-Specific Expression of *H. halys* Receptors

**FIGURE 4:**
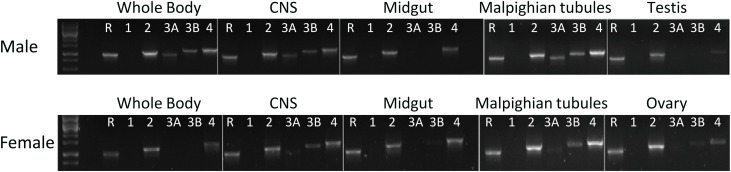
Expression of the PK/CAPA receptors in whole body and various organs of male and female in *Halyomorpha halys* as determined by RT-PCR. R, reference gene (GAPDH); 1, HalhaPK-R1a/b; 2, HalhaPK-R2; 3A, HalhaPK-R3a; 3B, HalhaPK-R3b; and 4, HalhaCAPA-R.

RT-PCR results revealed that *H. halys* GPCRs were differentially expressed in male and female adult tissues (Figure 4). Interestingly, HalhaPK-R1a and -R2b were both cloned from initial preps of cDNA made from RNA isolated from male whole bodies, however, they were not detected in any of the samples tested in RT-PCR experiments, despite repeated attempts using cDNA preps from multiple RNA isolations. HalhaPK-R2 was detected in all tissues tested, with the highest expression being in the male and female Malpighian tubules. HalhaPK-R3a and b were primarily detected in the Malpighian tubules and central nervous system. Varying expression levels of HalhaCAPA-R were detected in all tissues tested, with the highest expression observed in the Malpighian tubules.

### Age-Specific Expression of *H. halys* Receptors

**FIGURE 5:**
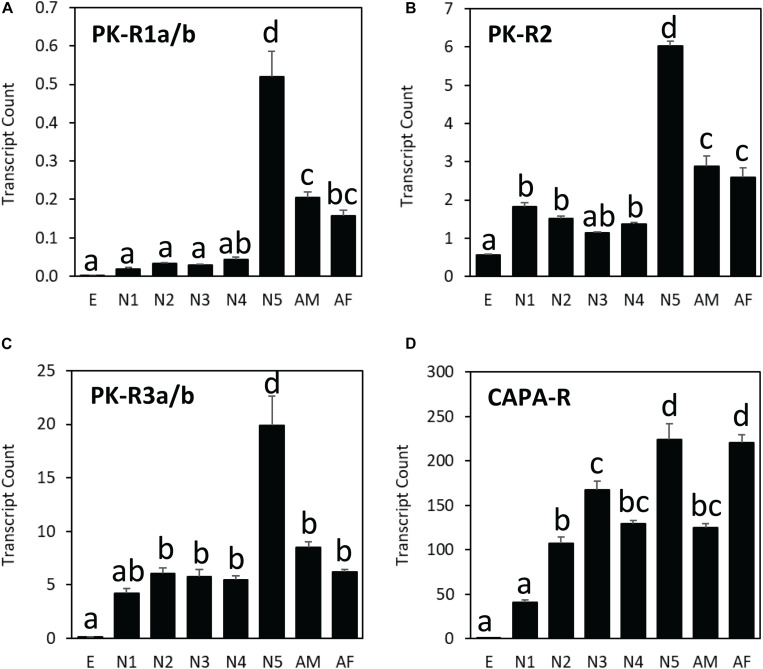
Transcript levels of **(A)** HalhaPK-R1a/b, **(B)** HalhaPK-R2, **(C)** HalhaPK-R3a/b, and **(D)** HalhaCAPA-R receptor genes in different developmental stages of *Halyomorpha halys* as measured by quantitative PCR, expressed in terms of transcript copies per 1,000 Rpn2 transcripts. E, egg; N1–N5, 1st–5th nymphs; AM, adult male; AF, adult female (mean ± SEM). Different letters denote significant differences (*P*< 0.05) determined by One-way ANOVA multiple comparisons statistical analysis.

Among different life stages, all three PK receptor genes (HalhaPK-R1, -R2, and –R3) were expressed the most in the last nymphal stage (N5), whereas the CAPA receptor gene (HalhaCAPA-R) showed broad-range expression throughout the life cycle, except in the egg stage (Figure 5). Among different receptor genes, HalhaCAPA-R was most highly expressed (50–250), followed by HalhaPK-R3s (5–20), HalhaPK-R2 (1–6), and HalhaPK-R1s (0–0.5). The three PK receptor genes did not have sex-biased expression, but the HalhaCAPA-R was more abundant in adult females than in adult males (Figure 5).

## Discussion

G protein-coupled receptors (GPCRs) are readily identified from insect genome sequences based on the presence of a well conserved, seven-transmembrane-domain topography (Hewes and Taghert, 2001). We identified six GPCRs through sequence homology-based searches of *H. halys* genomic and transcriptomic databases. The GPCRs were functionally characterized using PK and CAPA peptides *in vitro*. Upon amplification of these receptors via PCR it was discovered that two PK receptors, HalhaPK-R1 and HalhaPK-R3, were transcribed as two alternatively-spliced variants, which is not unusual among PBAN/PK receptors in moths (Lee et al., 2012; Nusawardani et al., 2013). The “a” and “b” versions of the receptors were alternatively spliced at the C-terminal ends and physically resided in the last intracellular domain of the proteins (Figure 1). We did not find any variants of the CAPA receptor in this study, although an atypical and inactive variant of the CAPA receptor has been found in *R. prolixus* (Paluzzi et al., 2010).

*H. halys* PK and CAPA receptors are expected to respond to PK peptides encoded from the *PBAN/pyrokinin* gene, and CAPA-PVK peptides encoded from the *capa* gene, respectively. In this study, HalhaCAPA-R was clearly distinguished from the other five receptors. It responded strongly only to CAPA-PVK peptides containing the PRVamide conserved motifs in the C-termini, but had no response to PK type peptides containing the PRLamide conserved motifs in the C-termini. Also, CAPA-PVK peptides did not activate any of the other PK receptors tested in a concentration-dependent manner. The CAPA receptor was expressed in all tissues and life stages we tested in this study. In addition, the CAPA receptor expression levels were much higher than the other *H. halys* receptors. In fact, *capa* genes are ubiquitously found across invertebrate phyla including Arthropoda (Predel and Wegener, 2006) and CAPA peptides and their receptors are more evolutionarily conserved (Choi et al., 2017). As expected, such a high expression level of CAPA receptor observed in Malpighian tubules suggests CAPA peptides might be involved in antidiuretic processes in the brown marmorated stink bug as in other hemipteran insects (Paluzzi et al., 2010; Paluzzi, 2012).

In our experiments, we found five GPCRs, HalhaPK-R1a/b, HalhaPK-R2 and HalhaPK-R3a/b, that belong to the PK receptor group based on their sequence homologies and binding responses to PK and CAPA peptides, however, functional studies did not clearly differentiate them as PK1 (= DH) or PK2 (= PBAN) receptors. Although HalhaPK-Rs 1a/b, 2, 3a/b all responded to PK2 peptides, HalhaPK-R1a/b responded weakly to PK2 peptides and had very low expression in the body, suggesting they might be losing their biological relevance in *H. halys*. Meanwhile, HalhaPK-R2 was much more sensitive to PK2 than the PK1 peptide, suggesting it might play a role as the primary receptor for PK2 peptides.

From the first report of a PBAN receptor in *Helicoverpa zea* (Choi et al., 2003) to the recent report of *Ostrinia furnacalis* (Luo et al., 2019), many GPCRs for PK2 (= PBAN) peptides have been identified and functionally characterized in a variety of insect groups. However, PK1 receptors (PK1-R or DH-R) have been identified and characterized in only 9 species, including the tick, since the first *Drosophila* PK1-R was reported (Cazzamali et al., 2005). Interestingly, HalhaPK-R3a/b responded to both types of PK2 and CAPA-DH (= PK1) peptides, but notably, HalhaPK-R3b was the most sensitive to CAPA-DH among the six GPCRs. Therefore, the result suggests HalhaPK-R3 is the primary receptor for CAPA-DH (= PK1) in *H. halys*. Based on the responses of the *H. halys* GPCRs to peptide ligands, HalhaPK-R3 and HalhaCAPA-R can be activated by all PKs (PK1 and PK2s) or only CAPA peptides, respectively. Actually, these two receptors are the most dominant in the *H. halys* organs tested in this study. Although HalhaPK-R2 is expected to be the primary receptor for PK2 peptides, HalhaPK-R3b strongly responded to both PK1 and PK2 peptides, thus it may be an alternative receptor in *H. halys*. Previously, it was found that PK2 receptors are more promiscuous in other insects, responding to both PK2 and DH ligands, but insect DH receptors tend to respond specifically to DH type peptides (Olsen et al., 2007; Nusawardani et al., 2013). Other studies have found the opposite, that DH receptors cross reacted to the PBAN/PK2 and DH/PK1 (Jiang et al., 2014a, b). It is difficult to generalize; therefore, it is possible that the receptor sensitivities and specificities shown here for *H. halys* have evolved differently than in other previously studied insect groups.

We then examined the expression patterns of the mRNAs of four *H. halys* PK GPCRs in selected tissues of *H. halys* and compared them between male and female. RT-PCR results revealed that the expression patterns of *H. halys* receptors were similar between male and female, with one exception being that HalhaPK-R2 was relatively more abundant in the Malpighian tubules and central nervous system of males than in female adults (Figure 4). Like the CAPA receptor, HalhaPK-R2 is ubiquitously expressed in *H. halys* tissues. Interestingly, strong transcription of HalhaPK-R2 was detected in testis and ovary, indicating a potential function involving muscle contraction or nutritional metabolism in these reproductive organs. PK2 peptides were previously isolated from cockroaches, and demonstrated to stimulate the contraction of hindgut muscles (Holman et al., 1986). Our RT-PCR and qPCR results for certain receptors did not always correlate. It is possible that HalhaPK-R3a and -R3b genes separated or combined for two different PCR measurements. For example, in qPCR experiments HalhaPK-R1a & -R1b and HalhaPK-R3a & -R3b were all detected in both male and female whole body samples, however, in RT-PCR experiments HalhaPK-R1a & -R1b were not detected in male or female samples and HalhaPK-R3a & -R3b were not detected in female whole body samples. One possible explanation for the difference between RT-PCR and qPCR experiments is that the sizes of the amplicons differed significantly: in RT-PCR experiments the full-length open reading frames were amplified, whereas in qPCR experiments only ∼100 bp fragments were amplified. These results might suggest that the mRNAs are being selectively degraded or are present only as fragments in certain RNA preparations. Furthermore, expression levels of HalhaPK-R1a and -R1b variants were low or not detected at all in the tissues we tested despite being cloned from RNAs isolated from *H. halys*.

In this study, we identified six GPCRs that show varying response profiles to peptides produced from *pyrokinin* and *capa* genes in the brown marmorated stink bug. These receptors might have pivotal roles in the regulation of critical physiological processes that govern the development and survival of this insect. Now that these receptors have been identified and characterized, they can serve as biological targets in the development of *H. halys* specific pest control methods.

## Data Availability Statement

The raw data supporting the conclusions of this article will be made available by the authors, without undue reservation, to any qualified researcher.

## Author Contributions

S-JA, JC, and MC performed all the experiments and analyzed the data. RV and the other authors wrote and edited the manuscript.

## Conflict of Interest

The authors declare that the research was conducted in the absence of any commercial or financial relationships that could be construed as a potential conflict of interest.

## Footnotes

1 https://i5k.nal.usda.gov
