# Supplementary material for: Identification and Characterization of GPCRs for Pyrokinin and CAPA Peptides in the Brown Marmorated Stink Bug, Halyomorpha halys (Hemiptera: Pentatomidae)
Source: Front Physiol. 2020 May 29;11:559. doi: 10.3389/fphys.2020.00559 (PMC7274154; doi:10.3389/fphys.2020.00559)
Supplement: Supplementary file 1 [file Image_1.pdf]

Supplementary Figure S1

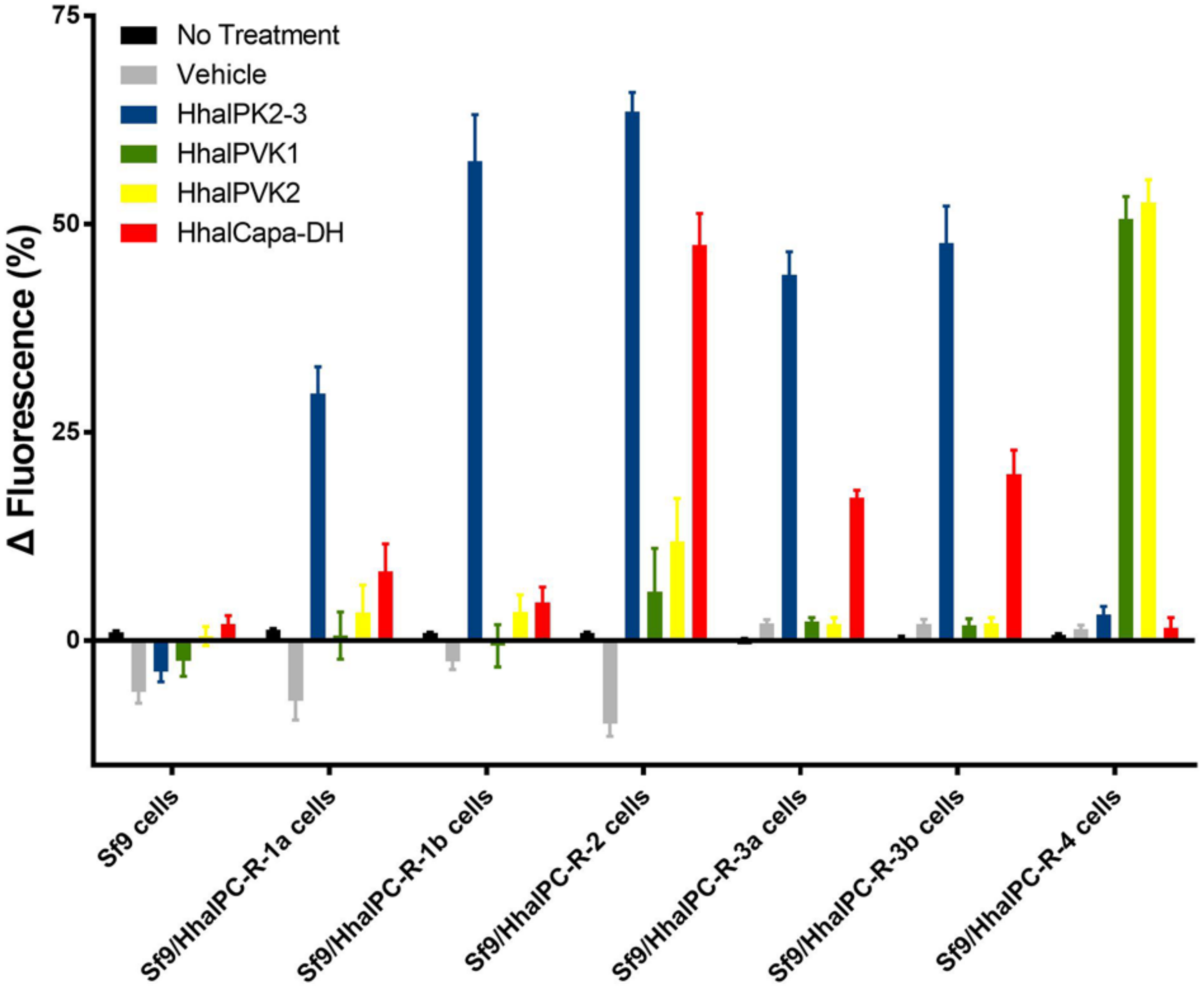

Figure S1. Responses of candidate *Halyomorpha halys* receptors to pyrokinin and CAPA peptides at 500 nM concentration. Data represent the mean ± SEM response of cells from three independent experiments.
