# Supplementary material for: Identification and Characterization of GPCRs for Pyrokinin and CAPA Peptides in the Brown Marmorated Stink Bug, Halyomorpha halys (Hemiptera: Pentatomidae)
Source: Front Physiol. 2020 May 29;11:559. doi: 10.3389/fphys.2020.00559 (PMC7274154; doi:10.3389/fphys.2020.00559)
Supplement: Supplementary file 2 [file Table_1.pdf]

Table S1. Primers used in this study. Kozak sequences are underlined. Amplicon sizes include Kozak sequences and stop codons in cases of cloning and RT-PCR.

Target gene	Usage	Amplicon size (bp)	Direction	Sequence (5'-3')
HalhaPK-R1a/b	Cloning RT-PCR	1380	forward	GCCACCATGGACGATTTTTCAGTGACAGATAGC
			reverse	CTACCTCGGGGGTTCTAGGAGCTCC
	qRT-PCR	117	forward	ACAACTACTCGGTGCTCAGC
			reverse	TCCTGAAGTTGAAGACGAGGG
HalhaPK-R2	Cloning RT-PCR	1176	forward	GCCACCATGGCTGACTCCACAGAGACG
			reverse	TCACCTGGAATTGTTGAGCTGACCCATG
	qRT-PCR	113	forward	GTGAGAGCCATCGACTCCAG
			reverse	GAAGCCAGTTCAGCTCCACT
HalhaPK-R3a	Cloning RT-PCR	1038	forward	GCCACCATGGAAGAACTGGAGTCTTACGAGTTCC
			reverse	TCAGGTACGTCCTCCGCTTCCTTTTCCG
HalhaPK-R3b	Cloning RT-PCR	1224	forward	GCCACCATGGAAGAACTGGAGTCTTACGAGTTCC
			reverse	TCACGCCTGGCGGACTAAGAGGTCTTGC
HalhaPK-R3a/b	qRT-PCR	103	forward	TGCTGTGGTAGTGGCATTCT
			reverse	GAAGGTTGAGCTGGGTGTGT
HalhaCAPA-R	Cloning RT-PCR	1383	forward	GCCACCATGAGCAGGAACATC
			reverse	TCAGATATAGGTTTCTTTTTCAGTATTTTGATTGG
	qRT-PCR	102	forward	TTCGGATGCTAGTTGCCGTT
			reverse	CGTAGTTGGGCCACGACTTA
HalhaRpn2 (housekeeping)	qRT-PCR	110	forward	GCCCAGCTTATCTCCGACAA
			reverse	TGTAGCAGCTTCCTGATGGC
HalhaGapdh (housekeeping)	RT-PCR	999	forward	ATGTCTAAAATTGGTATTAATGGATTTGGA
			reverse	TTAGTCTTTTGTTTGCATGTATCTGATAAG
